# Predictive typology of subjective quality of life among participants with severe mental disorders after a five-year follow-up: a longitudinal two-step cluster analysis

**DOI:** 10.1186/s12955-015-0346-x

**Published:** 2015-09-21

**Authors:** Marie-Josée Fleury, Guy Grenier, Jean-Marie Bamvita

**Affiliations:** Department of Psychiatry, McGill University, Douglas Hospital Research Centre, 6875 LaSalle Blvd, Montreal, QC Canada H4H 1R3; Douglas Hospital Research Centre, Montreal, QC Canada H4H 1R3; Montreal Addiction Rehabilitation Centre-University Institute, 6875 LaSalle Blvd., Montreal, QC Canada H2M 2E8

**Keywords:** Subjective quality of life, Predictors, Clusters, Severe mental disorders

## Abstract

**Background:**

This study aims to create a predictive typology of quality of life at five-year follow-up of 204 individuals with severe mental disorders, according to clinical, socio-demographic, and health service use variables.

**Methods:**

Participant typology was carried out by means of two-step cluster analysis. Independent variables were measured at T0 and subjective quality of life (SQOL) at T2.

**Results:**

Analysis yielded four classes. SQOL at T2 was higher than the mean in Class 4 (“Older, poorly educated single men living in supervised housing, with psychotic disorders but with few serious needs, receiving substantial help from services”) and lower than the mean in Class 2 (“Young females with serious needs and co-occurring mental and addiction disorders living in independent apartments”).

**Conclusion:**

Given that predictive SQOL varies in relation to combinations of associated variables, it would be useful for treatments or service programs to target specific predictors to the different profiles.

Quality of life is a key outcome indicator for the planning and evaluation of health services, primarily those for populations with chronic disorders, such as individuals with severe mental disorders [1–3]. Mental health studies on quality of life emerged when individuals with severe mental disorders began to be discharged from psychiatric hospitals and treated in the community [4, 5]. Interest in the impacts that poverty, housing, personal safety, loneliness and other socio-demographic variables have on mental health has also stimulated interest in quality-of-life research [6]. Improving the quality of life of individuals with severe mental disorders is now an objective for all new mental health programs, practices and services [7]. Indeed, poor quality of life is a strong predictor of relapse among individuals with schizophrenia [3], alcohol addiction [8, 9] and maybe other mental disorders.

There is an absence of consensus on the definition of quality of life [3, 10]. The earliest conceptualizations of quality of life among individuals with severe mental disorders focused on dimensions important for their community integration such as personal safety and psychosocial support [10]. Several new conceptual models of quality of life have emerged more recently, where quality of life for patients is defined in various ways: as the gap between individual expectations and achievements [11]; as the outcome of interactions among psychotic symptoms, medication side effects and psychosocial performance [12], or between an array of distress and protective factors [13]. Quality of life is also viewed as equivalent to reintegration into normal living [14] or improvements in lifestyle, greater autonomy and positive self-concept [15]. This proliferation of definitions suggests perhaps why multiple instruments have been designed to measure quality of life among mental health patients [10]. Moreover, while most of these instruments were expert-rated, others are self-report instruments, both of which may introduce a bias into the results [16].

Quality of life is, in fact, a heterogeneous concept [2] with objective and subjective dimensions. Objective dimensions refer to aspects related to the social environment and social functioning, while subjective dimensions include “well-being,” “happiness” or “life satisfaction”[1]. Several studies have shown that objective and subjective quality of life (SQOL) are not strongly correlated [5, 17–19]. The perception of SQOL in individuals with severe mental disorders is linked to multiple factors [20, 21]. Cross-sectional and comparative studies have found associations between a secondary diagnosis, such as a mood disorder [22–25] or an anxiety disorder [24], and lower SQOL, in individuals with schizophrenia or other psychotic disorders. A strong association between serious needs and lower SQOL has also been reported in the literature [26–28]. Socio-demographic variables are less strongly correlated with SQOL. However, some studies have found higher SQOL in females [29, 30], older individuals [30], individuals with a higher income [31] and individuals in the work force [32].

A number of longitudinal studies has investigated predictors of SQOL in individuals with severe mental disorders [17, 21, 33–44]. Those studies found that a reduction in the number of serious needs [34, 37, 40, 44], symptom severity [35, 36], anxiety [38] or substance abuse [21, 45] improves SQOL. A strong social network [21, 36, 40] and health service use [21] are other predictors of improved SQOL reported in the literature. However, while regression analysis used in longitudinal studies allows identification of predictors of SQOL, it is not possible to establish a typology of individuals according to their SQOL and other associated variables.

Cluster analysis is a useful method for establishing a typology of individuals with mental disorders [46]. This type of analysis allows them to be included in subgroups characterized by different profiles correlated with clinical variables (e.g., diagnoses, needs), socio-demographic variables (e.g., age, gender, civil status), and health service use (e.g., help received from services, continuity of care). Cluster analysis has previously been used to identify profiles of individuals with severe mental disorders [46] or common mental disorders [47], hospitalized for the first time [48], hospitalized with co-occurring severe mental and substance abuse disorders [49], frequent users of in-patient mental healthcare services [50, 51], individuals with schizophrenia treated in the community [52] and homeless people with severe mental disorders [53, 54]. However, to the best of our knowledge, cluster analysis has never been used to analyse predictors of SQOL.

Using cluster analysis, this study aims to create a predictive typology of quality of life at five-year follow-up among individuals with severe mental disorders, on the basis of clinical, socio-demographic, and health service use variables.

## Methods

### Study design and healthcare network characteristics

This prospective study involved individuals with severe mental disorders followed at a mental health university institute (MHUI), located in south-west Montreal (Québec, Canada). A total of 258,000 inhabitants live in this urban area. Healthcare for the population is covered by the MHUI and by two health and social service centres (HSSCs) created through the merger of general hospitals, community local health centres and nursing homes. The MHUI offers specialized mental health services (i.e., second-line and third-line services) while the HSSCs provide primary mental health services. Other professionals and organizations offering health services in this area have been identified in greater detail in previous publications [44, 55].

### Sample selection criteria and recruitment of the main sample

Inclusion criteria for participation in the study were: (1) age 18–65 years; (2) having been diagnosed with a severe mental disorder according to the DSM-IV (e.g., schizophrenia, mood disorders); (3) living in south-west Montreal; (4) being followed up by the MHUI at baseline; (5) allowing the research team to examine the participant’s medical record; and (6) agreeing to refer the research team to the participant’s principal case manager. For eligible candidates, case managers would subsequently be contacted and asked to complete a questionnaire on their patient’s functional status. Patients considered unable to complete questionnaires (i.e., clinically too fragile or unstable) were excluded from the study. Moreover, since the study had to assess quality of life among individuals with severe mental disorders transferred to, or living in, the community, people undergoing involuntary psychiatric treatment, as determined by a judicial board, and those hospitalized in inpatient services at the time of the recruitment were excluded from the study.

Baseline recruitment (T0) involved diverse strategies: posters displayed at the Douglas MHUI and in HSSCs for participant self-referral; recruitment at out-patient clinics; and information sessions or flyers explaining the project to mental healthcare providers or housing resources staff in the district. The research team worked closely with an advisory committee of decision makers from the mental health district to assist with data collection.

Participant data were collected from December 2008 to September 2010 (baseline, T0), from January 2011 to November 2011 (T1), and from June 2013 to April 2014 (T2). The 5-year study period was equivalent to the implementation period of the Quebec Mental Health Action Plan [56], a major reform of the Quebec mental healthcare system targeting the transfer of patients from institutional settings to the community where they would be followed by primary care services. The choice of conducting T1 follow-up at 2 years was to perform a “halfway evaluation”. The baseline window (T0) was 22 months, as we needed to enrol a large number of participants (at least 350). For TI and T2, participants were contacted consecutively, according to their entry into the study, such that data collection for each participant was spaced throughout a period of two to five years. The measurement period took a maximum of one year for T1 and T2 respectively.

**Fig. 1:**
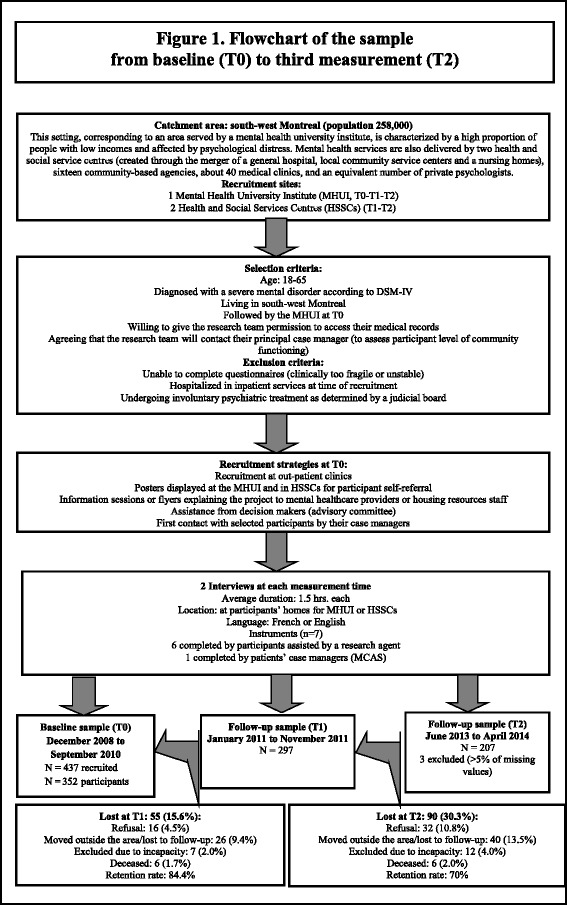
Flowchart of the sample from baseline (T0) to third measurement (T2)

At each measurement time, participants were met by trained clinical research agents to help them complete the instruments (except for the community functioning scale). Research agents maintained close contact with a research coordinator and the research team in order to maintain quality data processing. With the exception of self-referrals, the selected participants were first contacted by their case manager, who gauged their interest in participating in the study, and subsequently referred the participants to the research team. Each participant was required to sign a consent form after the study was described. Two 90-min interviews, held at one-week intervals, were conducted for each participant. The study protocol was approved by the ethics boards of the MHUI and the two HSSCs. Further details on sample selection criteria and recruitment of the main sample are shown in Fig. 1.

### Instruments

Data were collected from seven questionnaires, administered in either English or French, and from participants’ medical records at the MHUI. SQOL was assessed with the modified version of the Satisfaction with Life Domains Scale (SLDS), initially developed by Baker and Intagliata [4], translated into French and validated by Caron et al. [57]. Like other instruments such as the Lancashire Quality of Life Profile [58], the Quality of Life Interview Scale [59] or the Wisconsin Quality of Life Index [60], the SLDS is an expert-reported scale designed specifically for individuals with chronic mental disorders [3]. The SLDS assesses satisfaction in 20 life domains. Participants are asked to indicate their emotional state [61–63] by choosing from among seven stylized faces and rating each on a seven-point scale, ranging from the saddest face (score 1) to the happiest face (score 7). The item scores were added up to arrive at a total score. The psychometric properties of the SLDS are good. The internal consistency of the overall scale is excellent (Cronbach’s alpha of 0.84 according Baker and Intagliata [4] and of 0.92 for the French version validated by Caron [57]), and the temporal stability of the SLDS proved good (*r* = 0,73; [57]) which makes it an effective instrument for measuring SQOL. The SLDS is among the scales that were included in their entirety in the Wisconsin Quality of Life Index, a multidimensional instrument containing 113 items [60, 64].

Other questionnaires used were (1) the Multnomah Community Ability Scale (MCAS), assessing patient functional status in the community (e.g., obstacles to functioning, social competence; CA: 0.87) [65]; (2) the Social Provisions Scale (SPS), exploring patient levels of social integration and support (e.g., reassurance about one’s value, the need to feel useful; CA: 0.92) [66]; (3) the Montreal Assessment of Needs Questionnaire (MANQ), measuring the number and severity of need, and the level of help received from relatives or from services [67]; (4) the Alcohol Use Disorders Identification Test (AUDIT), evaluating alcohol consumption levels and consequences (CA: 0.88) [68]; (5) the Drug Abuse Screening Test-20 (DAST-20), assessing patient drug use and consequences (CA: 0.74) [69]; and (6) the Alberta Continuity of Services Scale for Mental Health (ACSS-MH), measuring service continuity (e.g., system access, team function; CA: 0.78 to 0.92) [70]. All questionnaires were completed by the participants, except for the MCAS, which was completed by their principal case managers.

### Analyses

Missing values were less than 5 % per variable and were replaced by mean values. Univariate and cluster analyses were carried out using SPSS version 21. Univariate analyses included frequency distributions for categorical variables, and mean values for continuous variables. The trend in SQOL (according to the SLDS score) from baseline (T0) to the second follow-up period (T2) was estimated by subtracting the mean value of the furthest time from that of the most recent time (SLDS score at T2 – SLDS score at T0). The overall objective was to obtain three groups with acceptable sample sizes that would insure sound statistical comparisons. The same cut-off method was used by Ruggeri et al. [6]. Participants with SLDS trend scores between −10 and +10 were considered “stable.” Those with scores lower than −10 were considered “deteriorated,” while those with scores higher than +10 were considered “improved.”

The participant typology was developed by means of TwoStep Cluster Analysis in the SPSS Statistics 21.0 package [71]. This analysis proceeds in two steps: pre-clustering of participants into small subclasses, followed by the final clustering of subclasses into an appropriate number of classes based on specific statistical tests, or into a number of classes determined by the statistician according to the optimal interpretation of the model. Cluster analysis is similar to latent class analysis (LCA) run with the SAS package. These two techniques were introduced in the 1950s [72, 73]. They are similar in that they are both designed to identify latent clusters of subjects with similar profiles. They generate mutually exclusive and exhaustive classes; and use an objective method to determine the number of classes (the Bayesian Information Criterion for LCA or the Schwarz Bayesian criterion for two-step clustering); they work with categorical and continuous variables. Cluster analysis has the advantage of being more widely known and used than LCA.

Variables selected were classified as continuous or categorical. Categorical variables measured at baseline (T0) were gender, education, sources of income, civil status, housing status, primary diagnosis: mood disorders and psychotic disorders (schizophrenia, schizophrenia spectrum disorders and delusion); and secondary diagnosis: anxiety disorders, and personality disorders. Continuous variables measured at baseline (T0) were: age, severity of needs (MANQ score), addiction to drugs and alcohol (DAST-20 score and AUDIT score), functional status in the community (MCAS score), social support (SPS score), level of help received from services or relatives (MANQ score), and service continuity (ACSS-MH score). The variables taken into consideration were those which, according to previous studies, could influence a change in SQOL in individuals with severe mental disorders [21, 30, 32, 33, 36, 40, 45]. The SLDS score, measured at T2, was the outcome variable. Categorical variables were entered first, followed by continuous variables. The log-likelihood method was used to determine inter-subject distance and specific classification of participants. The first model was produced using the Schwarz Bayesian criterion, yielding two classes. Many different models were subsequently produced using a different number of predetermined classes. The final number of clusters was set at four, according to their overall contribution to inter-class homogeneity, as determined by the diagnostic model improvement test.

## Results

Overall, 437 individuals were recruited at baseline (T0), with 352 (80.5 %) consenting to participate. Of the 352 participants enrolled at baseline (T0), 297 (84.4 %) were interviewed at T1 [45]. The 85 individuals who refused to participate were compared with the study participants with respect to age and type of housing. No statistically significant differences were found with respect to the following variables: (1) intermediary resource participants (Chi-square: 5.999 (*P* = 0.199)); (2) foster home participants (Chi-square: 4.482 (*P* = 0.482)); or (3) other types of housing (Chi-square: 3.229 (*P* = 0.665)). Participants were also compared with respect to gender distribution (total sample). No statistically significant difference was found (Chi-square: 1.210; *P* = 0.271). Of the 297 participants at T1, 207 (69.6 %) were interviewed at T2; 32 (10.8 %) refused to participate; 12 (4.0 %) were excluded due to incapacity; 6 (2.0 %) were deceased, and 40 (13.5 %) had moved away from the study area or were lost to follow-up. Of the 207 participants at T2, 3 were excluded because of the large amount of missing data. The retention rate after five years was thus 59 %. Comparison analyses using cross-tabulation on categorical variables showed no differences in terms of gender and education, either between T0 and T2 samples (gender: *X*^2^ = 0.001, *p* = 0.982; education: *X*^2^ = 0.606, *p* = 0.436) or between T1 and T2 samples (gender: *X*^2^ = 0.099, *p* = 0.754; education: *X*^2^ = 0.942, *p* = 0.344). Moreover, comparison analyses using one-way ANOVA on MCAS scores (community ability) at T0, T1 and T2 yielded no significant differences between the three measurement times (*p* = 0.406). Comparison analyses were also carried out between participants lost-to-follow-up and those remaining in the sample, with baseline characteristics for age (*p* = 0.174), and MCAS score (*p* = 0.533). No statistically significant differences were found.

Participant socio-demographic, clinical and health service use characteristics at baseline are displayed in Table 1. Males represented 52 % of the sample. The mean age was 51 years. Only 40 % had some post-secondary education. More than 50 % of the sample lived in independent apartments and 85 % were single. The main diagnoses were psychotic disorders (schizophrenia: 36 % of the participants, schizophrenia spectrum disorders: 14 %, delusion: 7 %; Total: 49 % of the participants) and mood disorders (42 %). Several participants had more than one mental disorder, the mean being 1.6 (s.d. = 0.9). Table 1 also shows participant characteristics according to deterioration, stability or improvement in relation to the SQOL score. Only 18.6 % (*n* = 38) of participants saw an improvement in their SQOL over time, while 41.7 % (*n* = 85) showed stability and 39.7 % (*n* = 81) experienced a deterioration. Females were more likely to experience improvements in their SLDS scores over time, compared with males, while older participants (50 yrs. and over) were more likely to report a deterioration in their SQOL, compared with younger participants (under 50 yr.). Deterioration in the SLDS score was found in participants living in supervised apartments or other types of supervised housing (group homes, foster homes), whereas stability was found in participants living in independent apartments. Participants with mood disorders and addiction to drugs and alcohol also experienced improvements in their SLDS scores, whereas those with psychotic disorders seemed to deteriorate, and those with personality disorders showed stability.

**Table 1:** Patient distribution according to deterioration, stability or improvement in SLDS scores from baseline to T2

	Variables at baseline		Total sample		Deteriorated		Stable		Improved	
			(*n* = 204)		(*n* = 81)		(*n* = 85)		(*n* = 38)	
			n/mean	%/SD	n/mean	%/SD	n/mean	%/SD	n/mean	%/SD
Socio-demographic variables	Age (n, %)	<40 yr.	43	21.1	16	19.8	18	21.2	9	23,7
		40–49 yr.	66	32.4	24	29.6	27	31.8	15	39.5
		>50 yr.	95	46.6	41	50.6	40	47.1	14	36.8
	Gender (n, %)	Female	98	48.0	38	46.9	38	44.7	22	57.9
		Male	106	52.0	43	53.1	47	55.3	16	42.1
	Civil status (n, %)	Other than single	30	14.7	10	12.3	13	15.3	7	18.4
		Single	174	85.3	71	87.7	72	84.7	31	81.6
	Education (n, %)	Primary/Secondary school	123	60.3	53	65.4	49	57.6	21	55.3
		College/University	81	39.7	28	34.6	36	42.4	17	44.7
	Sources of income (n, %)	Welfare	129	63.2	59	72.8	49	57.6	21	55.3
		Other	75	36.8	22	27.2	36	42.4	17	44.7
	Type of housing (n, %)	Independent apartment	121	59.3	40	49.4	59	69.4	22	57.9
		Supervised apartment or other types of supervised housing	83	40.7	41	50.6	26	30.6	16	42.1
	SPS scores^a^ (mean, SD)		71.0	6.6	70.9	5.7	71.4	6.7	70.6	8.1
Clinical variables	Severity of needs^b^ (mean, SD)		43.3	29.5	40.5	28.0	44.1	28.6	47.3	34.8
	MCAS score^c^ (mean, SD)		65.6	10.0	64.1	10.5	67.2	9.0	65.3	10.4
	Primary diagnosis (Severe mental disorders)									
	Mood disorders (n, %)		86	42.2	24	29.6	38	44.7	24	63.2
	Psychotic disorders^d^ (n, %)		100	49.0	50	61.7	38	44.7	12	31.6
	Secondary diagnosis									
	Anxiety disorders (n, %)		25	12.3	7	8.6	14	16.5	4	10.5
	Personality disorders (n, %)		58	28.4	14	17.3	34	40.0	10	26.3
	Addiction^e^ (mean, SD)		8.5	7.3	7.9	6.6	8.6	7.7	9.4	8.0
	Number of mental disorders (mean, SD)		1.6	0.9	1.5	0.9	1.7	0.8	1.7	0.9
Health service use and appreciation	Help from relatives^f^ (mean, SD)		23.0	23.0	19.1	20.4	25.4	25.0	25.9	23.1
	Help from services^g^ (mean, SD)		35.2	21.3	36.4	22.5	34.0	19.8	35.5	22.1
	ACSS-MH score^h^ (mean, SD)		122.1	13.3	123.2	11.1	122.1	9.5	119.8	22.2

^a^Social Provisions Scale (SPS) score: Min = 24, Max = 96; Higher = positive

^b^Severity of needs (MANQ score): Min = 0; Max = 260; Higher = negative

^c^Multnomah Community Ability Scale (MCAS) score: Min = 17; Max = 85; Higher = positive

^d^Psychotic disorders = schizophrenia + schizophrenia spectrum disorders + delusion

^e^Addiction = Alcohol Use Disorders Identification Test (AUDIT) score + Drug Abuse Screening Test-20 (DAST-20) score

^f^Help form relatives (Montreal Assessment of Needs Questionnaire (MANQ) score); Min = 0; Max = 260; Higher = positive

^g^Help from services (MANQ score); Min = 0; Max = 260; Higher = positive

^h^Alberta Continuity of Services Scale for Mental Health (ACSS-MH) score: Min: 24; Max: 168; Higher = positive

Table 2 displays the four classes of participants generated from the two-step cluster analysis. The cluster model retained twenty-three variables based on their importance for the characterization of the participants and their discriminative results between clusters. This process led to the elimination of the functional status in the community (MCAS) score, the social support (SPS) score, and help received from relatives (MANQ) score, all of which had yielded very similar mean scores among clusters.

**Table 2:** Predictive typology of subjective quality of life among participants with severe mental disorders: A longitudinal two-step cluster analysis

		Classes				
		1	2	3	4	Combined
		*n* = 49 (24.0)	*n* = 45 (22.1)	*n* = 48 (23.5)	*n* = 62 (30.4)	*n* = 204 (100)
Age categories at T0 [n(%)]	<40 yr.	6 (14.0)	14 (32.6)	10 (23.3)	13 (30.2)	43 (100.0)
	40–49 yr.	12 (18.2)	19 (28.8)	26 (39.4)	9 (13.6)	66 (100.0)
	50 yr. and over	31 (32.6)	12 (12.6)	12 (12.6)	40 (42.1)	95 (100.0)
Gender at T0 [n(%)]	Females	33 (33.7)	29 (29.6)	15 (15.3)	21 (21.4)	98 (100.0)
	Males	16 (15.1)	16 (15.1)	33 (31.1)	41 (38.7)	106 (100.0)
Education at T0 [n(%)]	Secondary school or less	11 (8.9)	33 (26.8)	17 (13.8)	62 (50.4)	123 (100.0)
	College or over	38 (46.9)	12 (14.8)	31 (38.3)	0 (0.0)	81 (100.0)
Sources of income at T0 [n(%)]	Welfare	12 (9.3)	25 (19.4)	38 (29.5)	54 (41.9)	129 (100.0)
	Other	37 (49.3)	20 (26.7)	10 (13.3)	8 (10.7)	75 (100.0)
Civil status at T0 [n(%)]	Non-single	19 (63.3)	7 (23.3)	4 (13.3)	0 (0.0)	30 (100.0)
	Single	30 (17.2)	38 (21.8)	44 (25.3)	62 (35.6)	174 (100.0)
Housing status at T0 [n(%)]	Independent apartment	34 (28.1)	45 (37.2)	28 (23.1)	14 (11.6)	121 (100.0)
	Supervised apartments or other types of supervised housing	15 (18.1)	0 (0.0)	20 (24.1)	48 (57.8)	83 (100.0)
Mental disorders at T0	First diagnosis (Severe mental disorders)					
	Mood disorders [n(%)]	42 (48.8)	28 (32.6)	5 (5.8)	11 (12.8)	86 (100.0)
	Psychotic disorders^a^ [n(%)]	0 (0.0)	0 (0.0)	48 (48)	52 (52.0)	100 (100.0)
	Second diagnosis					
	Anxiety disorders [n(%)]	13 (52.0)	6 (24.0)	3 (12)	3 (12.0)	25 (100.0)
	Personality disorders [n(%)]	5 (8.6)	32 (55.2)	3 (5.2)	18 (31.0)	58 (100.0)
	Addiction^b^ [mean (SD)]	6.7 (5.1)	10.9 (11.0)	8.9 (6.7)	7.7 (5.4)	8.5 (7.3)
Severity of needs^c^ at T0 [mean (SD)]		40.7 (29.4)	61.4 (35.0)	43.2 (26.2)	32.1 (20.9)	43.3 (29.5)
Help from services^c^ at T0 [mean (SD)]		29.1 (18.0)	33 (19.7)	37 (18)	40.3 (25.6)	35.2 (21.3)
ACSS-MH^d^ score at T0 [mean (SD)]		116.2 (19.2)	123.4 (10.4)	124 (11.1)	124.4 (9.3)	122.1 (13.3)
Subjective quality of life (SLDS) score at T2 [mean (SD)]		99.1 (18.9)	89.9 (20.0)	97.9 (14.6)	106.7 (16.2)	99.1 (18.3)

^a^Psychotic disorders = schizophrenia + schizophrenia spectrum + delusion; ^b^Addiction = AUDIT + DAST-20; ^c^: Montreal Assessment of Needs Questionnaire (MANQ) score; ^d^ = Alberta Continuity of Services Scale for Mental Health

Class 1: “Highly functional older females having mood and anxiety disorders receiving little help from services and with little continuity of access to services but with a mean SQOL”

Class 2: "Young females with serious needs and co-occurring mental and addiction disorders living in independent apartments and with a very low SQOL”

Class 3: “Middle-aged males, well-educated, but poor and single with psychotic disorders and a mean SQOL”

Class 4: “Older, poorly educated single males living in supervised apartments or in other types of supervised housing, with psychotic disorders but few serious needs, receiving a large amount of help from services and with a very high SQOL”

Class 1 contained a higher proportion of participants who were older, female, who had a higher level of education, and who had sources of income other than welfare (i.e., employment income). This class had the highest proportion of individuals with mood and anxiety disorders, and no individuals with psychotic disorders. People in this class had the lowest ACSS and addiction scores (AUDIT and DAST), and the least amount of help from services. They ranked second in terms of SQOL score at T2. This class was labelled as “highly functional older females having mood and anxiety disorders receiving little help from services and with little continuity of access to services but with a mean SQOL.”

Class 2 contained a higher proportion of participants who were younger, female, less educated, mostly affected by personality disorders, mood disorders and addiction, and all living in independent apartments. People in this class had the highest severity of needs scores and the lowest SQOL scores at T2. They ranked second in terms of mood disorders. This class was labelled as “young females with serious needs and co-occurring mental and addiction disorders living in independent apartments and with a very low SQOL.”

Participants in Class 3 were mostly middle-aged (40 to 49 yr.), predominantly male, and well educated, but living on welfare. The majority lived alone. Most of them had psychotic disorders. This class had the third highest SQOL scores at T2. This class was labelled as “middle-aged males, well-educated, but poor and single with psychotic disorders and a mean SQOL.”

Class 4 was composed mainly of older participants (50 yr. and over). They were all single and poorly educated, and the majority were males receiving welfare and living mostly in supervised apartments or in other types of supervised housing. The highest proportion of people with psychotic disorders was in this class. They had the highest ACSS-MH scores, the highest amount of help from services, and the lowest severity of needs scores. They ranked first in terms of SQOL at T2. This class was labelled as “older, poorly educated single males living in supervised apartments or in other types of supervised housing, with psychotic disorders but few serious needs, receiving a large amount of help from services and with a very high SQOL.”

## Discussion

The proportion of participants who saw improvements (18.6 %) in their SQOL after five years was lower in the present study than the 26.4 % reported in the study by Ruggeri et al. [6]. Moreover, the proportion of individuals who saw deterioration in their SQOL was higher than that found in Ruggeri et al. [6] (39.7 % vs 19.8 %). Differing study timeframes (five-year vs two-year follow-up) and choice of instruments (the SLDS vs the Lancashire Subjective Quality of Life Profile [58]) may explain these differences. Moreover, our study was conducted during a major reform of the Quebec mental healthcare system [74]. Under Quebec’s 2005–2010 Mental Health Action Plan, patients living in specialized psychiatric services had to be assessed, and those deemed sufficiently stable were identified and transferred to the primary care sector in order to facilitate their integration into the community. This reform may very well have affected some participants, and may explain deterioration in their SQOL.

Four profiles emerged from the cluster analysis. In one of them (Class 4), the SQOL was higher than the mean at T2; in another one (Class 2), SQOL at T2 was lower; and in the last two (Class 1 and Class 3), SQOL scores at T2 were at the mean. Classes 2 and 4 did not share any of the variables included in the cluster analysis, except for education level, civil status and continuity of care. Interestingly, a large majority of the participants included in the cluster with the highest SQOL (Class 4) lived in supervised apartments or other types of supervised housing, were over the age of 50 years, were single and poorly educated, and had psychotic disorders. A possible explanation for these high scores may be that these individuals were satisfied with living in supervised apartments or other types of supervised housing and had consequently lowered their life expectations [1, 75]. Moreover, given that they all lived in supervised apartments or other types of supervised housing, Class 4 participants received more help from services and benefitted from greater continuity of care. Access to a regular source of healthcare is strongly associated with service use [76] and helps build a good therapeutic alliance between individuals with severe mental disorders and health professionals [77]. This situation often leads to a reduction in unmet needs and an improvement in health outcomes. Not surprisingly, the severity of needs among Class 4 participants was much lower than that in the other classes. According to the literature, the absence of serious needs is the most important predictor of a high SQOL in individuals with severe mental disorders [26, 39]. Furthermore, individuals with psychotic disorders, more particularly schizophrenia, often have a higher SQOL than those with mood disorders or other mental disorders [6, 75], even though their living conditions and life circumstances may be more adverse [75, 78]. This perception may, however, also be the result of poor insight and affective blunting among this clientele [75], or an overestimate of their level of functioning [79].

Conversely, Class 2 contained a higher proportion of personality disorders and addiction, and the second highest proportion of mood and anxiety disorders. It is recognized that individuals with personality disorders are more likely to have a lower SQOL than those affected by other mental disorders [80]. They are also more likely to have a greater number of unmet needs, mainly in the areas of self-care, psychotic symptoms, psychological distress, safety to self, safety to others, alcohol use, sexual expression, and money [81]. Individuals with co-occurring addiction and mental disorders are also very likely to experience unmet needs and are less likely to view their treatment as effective in comparison with those who have mental disorders only [82, 83]. Unlike the Class 4 participants, who lived mainly in supervised apartments or other types of supervised housing, all the Class 2 participants lived in independent apartments. Given that this type of housing offers less access to services, individuals living in independent apartments are required to interact more with their social environments in order to reduce their isolation and improve their SQOL [17].

Like the Class 2 participants, those in Class 1 were mainly females receiving little help from services. Moreover, no participant in these two classes was affected by psychotic disorders. The main differences between Class 1 and Class 2 emerged in the areas of diagnosis, severity of needs, age, education level, sources of income and civil status. Class 1 participants were more affected by mood disorders and anxiety disorders. However, they were less affected by addiction and personality disorders, which may explain their higher SQOL. Moreover, the Class 1 participants were older, better educated, non-single and likely to have better incomes, all variables associated with a higher SQOL [30–32]. They were also less likely than the Class 2 participants to be suffering from loneliness and isolation.

Like Class 4 participants, those in Class 3 were mainly male, single, receiving welfare as a source of income, and affected by psychotic disorders. The main differences between Class 3 and 4 were in the areas of education level, age, type of housing and severity of needs. Unlike the Class 4 participants, a large majority of the Class 3 participants had pursued a post-secondary education. The fact that most of the Class 3 participants received welfare benefits as their source of income may thus have been a major source of frustration, which could account for both their higher severity of needs and their lower SQOL. Individuals with severe mental disorders living in the community are often victims of stigma in the job market [84]. It is also possible that the individuals with higher education, mainly those who were middle-aged, were more aware of their rights and had difficulty accepting jobs and social conditions below their educational status. Finally, it is possible that, like the Class 2 participants, those in Class 3 living in independent apartments lacked the ability to interact with their social environments.

### Limitations

Our study had a few significant limitations. First, given that the number of variables introduced into the cluster analyses was limited, our results would not be generalized to other samples or populations if a different model were used. Second, because our sample represented a heterogeneous group of individuals with severe mental disorders, the results may not be applicable to a sample composed exclusively of individuals with psychotic or mood disorders. Third, as the SLDS is an expert-reported instrument, it is possible that some results were interviewer biased [16]. Fourth, the judgment of some participants may have been affected by their mental state at the time of the interview, possibly resulting in distorted responses on perceived SQOL [75]. Finally, a large number of participants were lost to follow-up, mainly at T2.

## Conclusions

To the best of our knowledge, this study is the first to establish a predictive typology of SQOL in individuals with severe mental disorders. Using cluster analysis was also innovative in that it included socio-demographic, clinical and health service use variables at T0, along with SQOL measured at a five-year interval (T2). Four different profiles were identified, showing considerable heterogeneity in SQOL after a five-year follow-up, mainly in terms of age, gender, diagnoses, type of housing and severity of needs. Given that predictive SQOL varies in relation to different combinations of socio-demographic, clinical and health service use variables, it would be useful for programs to target predictors specific to the various profiles. The results also show that SQOL at five-year follow-up was particularly low in the class that included more cases of co-occurring mental disorders and addiction and greater severity of needs (Class 2).

Our study highlights the need for mental health service planning and delivery to be better adapted to client profiles in order to improve SQOL. According to the severity of substance abuse problems, priority should be given to the provision of a variety of different services or interventions, including integrated dual disorder treatment, harm reduction or self-help groups such as Alcoholic Anonymous. Concerning individuals in Class 1, who were affected mainly by mood and anxiety disorders and exhibited low severity of needs, access to a regular source of healthcare, such as a family physician, could be sufficient to improve or maintain their SQOL. For individuals in Class 3, who have a high level of education but live on welfare, access to a job integration program seems to be a priority in order to improve their SQOL. Finally, our results show that supervised apartments or other types of supervised housing are adequate for older individuals with psychotic disorders, singles and those with low education, such as the participants in Class 4. Residential services matching the level of functioning of individuals with severe mental disorders should be maintained or developed.
